# The Prince of Wales's Hospital Fund

**Published:** 1899-01-21

**Authors:** 


### THE PRINCE OF WALES'S HOSPITAL FUND.

### MORE CRITICISM.

"A HOSPITAL SECRETARY" has renewed his attack on the mode of distributing the money at the disposal of the Council of this Fund. He publishes a table, in which he states that the grants to two general and two special hospitals reveal "the fallacious and partial basis of the awards of this Fund." By placing the award in one column and then multiplying what he gives as the cost per bed per annum by the number of beds to be opened, he makes out that the hospitals have to supply the following deficits: The Great Northern Central Hospital, one of £300; the London Temperance Hospital, one of £640; the City of London Hospital for Diseases of the Chest, one of £350; and the North London Hospital for Consumption, one of £305. These deficits are largely imaginary, and have little or no basis in fact, owing to the want of appreciation on the critic's part of the actual circumstances which govern the cost of an additional bed when opened in a hospital already fully-equipped and at work.

Besides this, however, "A Hospital Secretary" states that his figures are taken from "Burdett's Hospitals and Charities for 1898." It is not a little remarkable to find that this statement is erroneous, and that the figures in the table he quotes are not "taken from 'Burdett's Hospitals and Charities, 1898.'"

"A Hospital Secretary" exhibits great ignorance of hospital business on another point. He assumes in his calculations that the whole of the beds made available for patients will be continuously occupied throughout the whole year. It is, however, an admitted fact that in practice a deduction of about 20 per cent. must be made from the number of beds in order to arrive at the number which will be constantly occupied throughout the year. When this is done a calculation, based upon the cost per bed given by "A Hospital Secretary," wherever he may have obtained the figures from, shows that with the exception of the London Temperance Hospital the amount of the grants is adequate to meet the cost of maintenance, the actual difference varying from £44 to £90 on the total expenditure for the year, a sum which would probably disappear when the cost per bed is taken out at the end of the 12 months on the increased number of beds.

In the case of the London Temperance Hospital a deficiency is shown of £450, but, as Sir Savile Crossley has pointed out in his letter to the papers, the case of this institution was exceptional, because the hospital itself suggested that if the Fund would find half, a friend of the hospital would find the other half of the amount required to open 12 beds. This has been done, so that the London Temperance Hospital will in fact have £50 in hand on "A Hospital Secretary's" own calculation.

We said before, and we repeat, that "A Hospital Secretary" wisely concealed his name, because its publication in view of the above facts must have discredited him as a hospital secretary throughout the whole of the hospital world.

